# Society Meetings

**Published:** 1907-11

**Authors:** 


### SOCIETY MEETINGS.

At the Eighth District Branch meeting of the Medical Society of the State of New York, held at Buffalo, September 25th and 26th, 1907, a banquet was given at the Hotel Lenox on the evening of the first day. Among quite an important group of toasts was one responded to by Dr. Maud J. Frye of Buffalo.

Dr. Frye spoke as follows to the sentiment:

### WOMAN IN MEDICINE.

In responding to the toast Woman in Medicine, I can but feel as though expected to offer either explanation or apology for the existence of medical women. The line of thought which is suggested is one ordinarily absent from my mind. My problems in medicine are those you seek to solve. In the nature of things I am employed only by those patients who approve of or desire a woman physician and the work seems to me as natural a vocation for a woman as teaching or nursing.

The explanation of the invasion of the commercial, industrial and professional worlds by women within the last few decades, an invasion which so worries our strenuous President and whose anxiety is ponderously echoed from Princeton, lies, I think, in the fact that civilisation, education, science and invention have robbed women of their ancient occupations. There is a deeper reason than Mr. Roosevelt discerns behind race suicide. Not only do the times no longer demand the patriarchal families of pioneer days, but the domestic industries are also practically abolished. To spin and weave, to bake and brew in one's own household would in these days be extravagance instead of thrift. Whether the former days were better than these or not, they are gone, not to return.

"The old order changeth, yielding place to new."

By and by when women find themselves we shall see more good than evil coming from this evolutionary process. Obliged to look outside the home for occupation, women have tried their powers and are still trying to learn what they are equal to, what the world by granting them success, will approve of their doing. To my mind they will always best succeed in those callings which are allied to the race—long duties of child-rearing, teaching, nursing and home-making. I think in medicine we may say that judgment has been awarded.

In a recent address before the London School of Medicine for Women, Professor Osler gave it as his opinion that the profession of medicine, in spite of its difficulties and trials, is a satisfactory one for women; and according to the same high authority the several departments of medicine in which women will be most successful are the scientific branches of bacteriology, histology and pathology, institutional work, the mission field, school inspection, and, most important of all, general practice. In this women are specially fitted for dealing with the diseases of women and children. Concerning women in general practice he wisely remarks—that only those are fitted for it who among all the worries, anxieties and troubles can come up smiling every time.

Many people fail to appreciate the value of a medical education as a preparation for life. She who studies medicine, whether or not she becomes a successful physician, can hardly fail to be a kinder, saner and braver woman. I do not know that I can point to any great thing in medicine that women have done. I think that woman's genius lies rather in doing small things well. I do not now recall the names of more than one or two who have signally distinguished themselves. It is my privilege to know intimately many good women who are good physicians. One may speak more freely however, of the dead than of the living, and I wish to call to your memory tonight she who has lately gone from among us, Electa B. Whipple, a woman who came nearer to the ideal medical woman than any other it has been my fortune to know. Her intellectual and professional attainments were such as to command the respect of her confreres. Her life was lived on so high a plane that everything low and narrow and unworthy was shamed in her presence, yet her sanity and keen sense of humor made her one of the most delightful of comrades, as her serene nature and unfailing kindness made her the truest of friends.

So long as such women are found in the profession of medicine and so long as we other women hold them as examples for ourselves there will be no need for explanation or apology for our character or our work.

---

The Eighth District Branch of the Medical Society of the State of New York at its recent annual meeting held in Buffalo elected the following named officers for the ensuing year: president, A. D. Lake, Gowanda; first vice-president, E. E. Snow, Batavia; second vice president, J. W. Grosvenor, Buffalo; secretary, Lee Masten Francis, Buffalo; treasurer, Charles A. Wall, Buffalo.

---

The Medical Society of the County of Erie at its recent annual meeting held at Buffalo, October 14, 1907, elected officers for the ensuing year as follows: president, Edward Clark; vice presidents, C. A. Wall and Grover Wende; secretary, Franklin C. Gram; treasurer, A. T. Lytle; board of censors, Henry R. Hopkins, DeLancey Rochester, W. D. Greene, Francis E. Fronczak,

J. H. Grant; chairman of the committee on legislation, A. A. Hubbell; chairman of the committee on public health, Grover Wende; president of the committee on membership, T. H. McKee; delegates to the medical society of the State of New York, Drs. Brown, Heard, Thornton and O'Gorman; delegates to the eighth district branch, Drs. Greene, Rice, Wall, Fronczak and Durham.

---

THE Buffalo Academy of Medicine held meetings during the month of October as follows:

*Section on Surgery.*—Tuesday evening, October 1. Program: (*a*) Report of case of cerebral tumor simulating gumma and exhibits of blood specimens of various types of malaria found in the Philippines, Captain L. T. Hess, M. D., Fort Porter; (*b*) Traumatic paralysis of the third nerve, with report of case, Lee Masten Francis; (*c*) Intestinal obstruction, with report of cases, J. B. Young.

*Section on Pathology.*—A Stated Meeting was held Tuesday evening, October 15. Program (*a*) The viability of the typhoid bacillus under normal conditions, H. D. Pease, Albany, N. Y.; (*b*) Multiple epitheliomatosis; report of case, A. E. Woehnert.

*Section on Obstetrics and Gynecology.*—Tuesday evening, October 22. Program: (*a*) Lithopedion,—with report of cases, Herman E. Hayd; (*b*) Our friend the enemy, T. H. McKee.

---

THE Medical Society of the County of Wyoming at its annual meeting held at Warsaw, October 24, 1907, elected the following named officers for the ensuing year: president, Mary Greene, of Castile, vice president, J. L. Wright of Perry; secretary and treasurer, L. H. Humphrey of Silver Springs.

---

AT the annual meeting of the Medical Society of the County of Genesee, held at Batavia, October 2, 1907, the following named officers were elected for the ensuing year: president, H. E. Ganiard, of Stafford: vice president, Frank A. Miller, of Batavia; secretary and treasurer, George W. Cottis of Batavia; delegates to the eighth district meeting, Scott W. Skinner, of Le Roy and Ward B. Whitcombe, of Batavia; delegate to the medical society of the State of New York, E. E. Snow of Batavia.

Dr. Joseph Palmer, medical inspector of the public schools of Syracuse, read a paper upon Medical inspection of school children.

---

THE Medical Society of the County of Ontario at its annual meeting held at Canandaigua, October 8, 1907, elected officers for the ensuing year as follows: president, C. P. Merritt, of Clifton Springs; vice president, E. B. Sayre, of Allen's Hill; secretary and treasurer, D. A. Eiseline, of Shortsville; censors, S. B. Mead, F. E. McClellan, and J. H. Pratt; delegate to State Medical Society, A. L. Beahan; alternate, J. H. Jewett; delegate to district branch, George Means; alternate, W. B. Clapper. The fiftieth year of membership of Dr. L. F. Wilbur, Honeoye, was appropriately observed at the banquet which followed the business meeting.

---

The Medical Society of the County of Steuben, held its semiannual meeting at Corning, October 8, 1907. The following named officers were elected for the ensuing year: president, Charles O. Green, of Hornell; vice president, H. B. Smith, of Corning; secretary and treasurer, W. W. Smith, of Avoca.

---

The New York and New England Association of Railway Surgeons will hold its seventeenth annual meeting at the Academy of Medicine, New York, November 14-15, 1907, under the presidency of Dr. T. H. Dana, of Cortland, N. Y. A program on subjects of unusual interest and practical value to the busy surgeon has been arranged.

Dr. Robert Dawbarn will deliver the Address on Surgery, on November 14, and on the following day will give a clinic demonstrating his method of sequestration.

---

The International Congress of Tuberculosis will hold its next meeting at Washington, D. C., September 21, to October 12, 1908, under the presidency of Frank Billings, of Chicago. The secretary-general is John S. Fulton, Washington. The preliminary announcement is an elaborate one covering scientific work for the entire three weeks of the session. It is altogether the most complete announcement of its kind that has yet been issued so far in advance of a scientific medical meeting.

The committee on the congress has enlisted the interest of the federal government. Seven of the governmental departments have signified their intention to participate in the congress, and have petitioned the congress of the United States for the necessary authority and means. These are the departments of state, of the treasury, of war, of the navy, of the interior, of agriculture, and of commerce and labor.

---

The Medical Society of the State of New York will hold its one hundred and second annual meeting at Albany, Tuesday and Wednesday, January 28 and 29, 1908, under the presidency of Frederic C. Curtis, of Albany. Wisner R. Townsend, of New

York, is the secretary. Members desirous of reading papers are requested to send titles to the chairman of the scientific committee, Dr. Leo H. Neuman, 194 State Street, Albany.

